# Progress in Surgery

**Published:** 1899-12-16

**Authors:** 


### PROGRESS IN SURGERY.

### TUMOURS.

**The Ætiology of Cancer.**—The causal influence of external biological factors in the production of cancer has been much studied of late, but the results of this study have as yet by no means solved the ætiological problem. The subject of the ætiology of cancer has also been studied from the other side, that of the essential vitality of epithelial cells and their reaction to various irritants, and interesting results obtained. Dr. Hektoen[1] has collated some striking observations on this side of the subject. For instance, Ljunggeen, a Scandinavian physician, found that he could preserve sterilised bits of human skin in sterile ascitic fluid for months, and that the cells retained their vitality. Successful transplantation was made with pieces kept in such sterile fluid for a month. Small pieces of the transplanted skin, removed at various intervals, showed a marked proliferation of epithelial cells in which many nuclear figures had occurred. The cells grew into the granulation tissue of the raw surface as well as on the surface. Again, according to Loeb, "if a small bit of epithelium is placed in the centre of the crust covering a defect in the skin, it begins to send out processes in all directions into the crust, the cells acting as separate organisms, independent of blood supply or nervous influence." Such manifestations of vitality as these closely resemble those met with in cancer. Dr. William Russell[2] gives an account of the work of Sanfelice and Roncali upon blastomycetæ as a cause of cancer. Russell described in 1890 bodies he had found in cancers, both intracellular and extra cellular, which he held to be blastomycetes, and provisionally called "fuchsin bodies." Since then the trend of opinion has been to regard these bodies, not as parasites, but as degenerative appearances. Morphological study was perfected by Ruffer and others, but experimental work alone could settle which view was correct. The work of the Italians mentioned above (and, it may be added, of Plimmer) are in favour of the parasitic view. Sanfelice found that he produced in the tissues of cats typical Russell's bodies by injecting pure cultures of saccharomyces neoformans. By inoculating the mammæ of a dog with blastomycetes which had previously passed through a number of dogs, he succeeded in producing tumours and retraction of the nipples, and secondary growths in the lymphatic glands, and death in ten months with marked cachexia. All the growths were typical adeno-carcinomata. Sanfelice could obtain no cultures from these growths, but Plimmer has obtained a medium in which the blastomycetes of human carcinoma will grow. Roncali, as a result of histological research and inoculation experiments, considers that the genetic connection between blastomycetes and malignant tumours is proved. Plimmer found such organisms in 1,130 out of 1,278 specimens of cancer he examined. Some interesting examples of accidental inoculations of cancer are given by Roswell Park in the "cancer number" of the *Practitioner*. In one case the inoculation occurred along the suture tracks after extirpating a cancerous ovary. In another cancer developed along the line of incision made for relieving tension after removal of a cancerous mamma, the same knife being used throughout the operation. Langenbeck injected a watery emulsion of cancer, mixed with defibrinated blood, into the femoral vein of a dog. Nodules were found in the lungs two months later, having the histological characters of the original growth.

**Prevalence of Cancer.**—There has been much discussion as to whether the yearly increasing mortality from cancer, as revealed in the official mortality returns of the registrars in Great Britain and America, can be taken as reliable evidence that the prevalence of cancer is really increasing. The editor of the *British Medical Journal*[4] has recently discussed the matter, and also Dr. J. F. Payne. These discussions emphasise the fact that "the correct handling of vital statistics is a very difficult art," and in the past various mathematical mistakes have been made by writers on the subject. Dr. Billings has stated that "the enumerators' returns for the whole country (United States) were in 1880 deficient about 30 per cent. in the number of deaths." Yet these records have been appealed to with confidence to prove the supposed increase of cancer. Another fallacy pointed out by Dr. Andrews[3] has been the assumption that cancer must have increased, because the ratio of cancer deaths to deaths from all causes has increased. But if, in 1860, one death from cancer occurred to 100 deaths from all causes, and in 1890, two deaths from cancer to 100 deaths from all causes, we cannot therefore conclude that cancer has doubled during the 30 years interval. Obviously some or all of the apparent increase of cancer might be due to a diminution in the deaths from all causes, and the above ratios be as 1 in 100 to 1 in 50. Other errors are due to the fallacy of accepting the mean age at death as a test of the duration of life in a district. Another is dependent upon the failure to recognise the difference of significance between the death-rate from cancer per 1,000 living at all ages, and the death-rate from cancer at a particular age-group per 1,000 living at that particular age-group.

The statements recently made by Dr. Poore that he believed the increase of cancer was due to more people living to what may be called a cancerous age, and that made by Dr. Hutchinson that "cancer is increasing because more people are living long enough to die of it," are based upon statistics which the above fallacies vitiate. Dr. Newsholme pointed out in 1898 that there is still seen to be an enormous increase of registered mortality from cancer when the above fallacies are avoided, and the question chiefly in dispute is whether the whole of this increase is real, or whether a part is real and the remainder due to improved diagnosis, and still more improved certification of deaths.

It is an important fact that the increase of cancer mortality and the decrease of that from tuberculosis is similarly conspicuous in both England and America. Dr. Payne[5] concludes, from a careful study of statistics, that not only has there been an increase in the prevalence of cancer generally, but that the increase is specially noticeable in cancer of the digestive tract, and is very striking in the case of the tongue. It is roughly estimated that cancer of the tongue is increasing at a rate of nearly 4 per cent. annually. Further, cancer is increasing more rapidly in males than in females, and the increase is more marked in the later periods of life than in middle life. Payne reviews the causes which have been suggested for this increase. They are (1) Heredity: If cancer is hereditary and does not hinder fertility there must in every generation be an increasing number of persons possessing this liability. (2) Increased longevity: This explanation has no validity. The census returns of 1891 did not show that the proportion of persons above 45 years of age had at all increased in comparison with the censuses of 1871 and 1881. (3) The decline of tubercle: In England the reduction of the death-rate from phthisis is far more marked in the female sex, whereas the increase of cancer affects chiefly males. Moreover, in Ireland there has been no reduction of the mortality from phthisis, yet cancer has increased there. (4) Decline in zymotic diseases: There has been a very great decline in the mortality from these diseases; but we have seen above that the proportion of old people in the population has not increased, so we cannot say that more people are left to reach a cancerous age. (5) Prosperity: Mr. Roger Williams has in particular urged that cancer is a disease to which prosperous and well-nourished people are particularly liable. It is the poorer classes that, by their numbers, make national statistics, and there is no question that there has been an enormous improvement in the condition of these classes during the last 50 years. Williams suggests that the consumption of meat may be an important factor. Cancer is said, however, to be prevalent in India, where the diet is largely vegetarian. In this connection the increased frequency of cancer in the digestive tract should be remembered. (6) Alcoholism: There is no doubt that the average consumption of alcohol in this country has very greatly increased. Further, the highest mortality from cancer is found in the occupations of brewers, innkeepers, inn servants, and commercial travellers, and in all these occupations there is, no doubt, special temptation to indulge in alcoholic drinks. For special reasons, chimney-sweeps and copper miners are still more liable to cancer than any of the above. Amongst workers in agricultural districts the cancer mortality is much less. Town life itself may be one factor in the causation of cancer.

**Recurrence of Mammary Carcinoma.**—Mr. Simpson[6] has published the results of 100 cases of excision of the mamma in the practice of Mr. A. E. Barker. Of these cases 90 were of malignant disease. This includes 82 of scirrhus, four duct-cancers, two of colloid cancer, and two of scirrho-encephaloid. Five died as an effect of the operation, one each from pyæmia, septicæmia, cellulitis, syncope, and pleurisy. Of the four cases of duct-cancer all survived the three-year limit. Two are still alive at $10\frac{1}{4}$ and $7\frac{1}{2}$ years after the operation; another died of pyelitis $3\frac{3}{4}$ years after the operation without recurrence, and the other was lost sight of. Of the 85 malignant cases surviving operation 33 were alive three years after the operation, but 11 of these subsequently had recurrence, showing that Volkmann's three-year limit as a test of cure is unreliable; 43 of the cases died ultimately from recurrence, and the remaining six died from conditions of doubtful nature, but probably due to recurrence. Thus the percentage of cases which survived the three-year limit was 38·3. Of the 23 cases still living, and free from recurrence, one was operated upon 11½ years ago, one 10 years, one 6 years, two 5½ years, and one 5 years.

Heurtaux[7] publishes a case of sudden development of multiple and scattered cancerous growths in a woman, aged 77, who 30 years previously had had the left breast removed for carcinoma, together with the fat and glands of the axilla. During that interval she had been healthy. The nodular growths were situated about the old cicatrix on the chest and in the axilla, and also in the left pectoralis major, the left pleura, and the spinal column. Microscopically a nodule was proved to be carcinomatous. The recurrence was thus metastatic, and not in an organ or tissue the anatomical structure of which predisposed it to cancer. It looks as though special conditions, the nature of which is unknown, are required to permit of the growth of carcinoma, even in those who undoubtedly possess the germ of the disease.

[1] Annals of Surgery, June, 1899. [2] New York Med. Jour., July, 1899. [3] Brit. Med. Jour., July, 1899. [4] Lancet, Aug., 1899. [5] Lancet, Sept., 1899. [6] Lancet, July, 1899. [7] Brit. Med. Jour., June, 1899.

